# A quality assurance tool for daily checks of the gamma knife high definition motion management system

**DOI:** 10.1002/acm2.13099

**Published:** 2020-11-18

**Authors:** Robert Corns, Dhara Mehta, Whitney Massock, Leah Roberts

**Affiliations:** ^1^ Department of Radiation Oncology Brody School of Medicine East Carolina University Greenville NC USA; ^2^ Department of Physics East Carolina University Greenville NC USA

**Keywords:** Gamma Knife, HDMM, QA tool

## Abstract

**Purpose:**

This note describes the performance of a quality assurance (QA) tool built for daily checks of the Gamma Knife’s high definition motion management (HDMM) system.

**Methods:**

The tool is a three‐dimensional (3D)‐printed platform with a raised corner in the center. A reflector post is placed at the corner and the HDMM tool is zeroed to this position. Gage blocks produce very accurate gaps between the post and corner and the HDMM system’s readout is compared to the gage block thickness. The HDMM system and tool were tested for noise, stability, reproducibility, linearity, accuracy and overall setup times plus ease of use.

**Results:**

The QA tool performed with accuracies better than 0.1 mm. The setup and use of this tool take less than two minutes making it a suitable tool for daily use.

**Conclusion:**

This QA tool is a cost‐effective solution that provides a fast and easy confirmation of the HDMM accuracy, making it suitable for daily QA checks of the HDMM system.

## 1 INTRODUCTION

The high definition motion management system (HDMM) on the Gamma Knife Icon™ is an infrared stereo camera capable of tracking a reflective marker’s position with 0.1 mm accuracy.^1^, ^2^, ^3^, ^4^ A quality assurance (QA) program should check the accuracy on a routine basis. This requires a primary standard that is both accurate and precise and exceeds the HDMM system’s accuracy. Optical stages have been used^5^, ^6^ that move with an accuracy within 0.010 mm using piezoelectric motors or manual micrometer drives. Knutson developed a multipurpose phantom^7^ that measures spatial, temporal and latency properties of HDMM system. It operates remotely and can confirm the system response to exceeding the HDMM’s set threshold during treatment. These tools are used in monthly QA programs. Wu designed a 3D‐printed phantom^8^ that is suited for daily use. Their printer had submillimeter accuracy but could not consistently print a 2‐mm thick shim. Each shim had its thickness verified by a micrometer.

**Fig 1:**
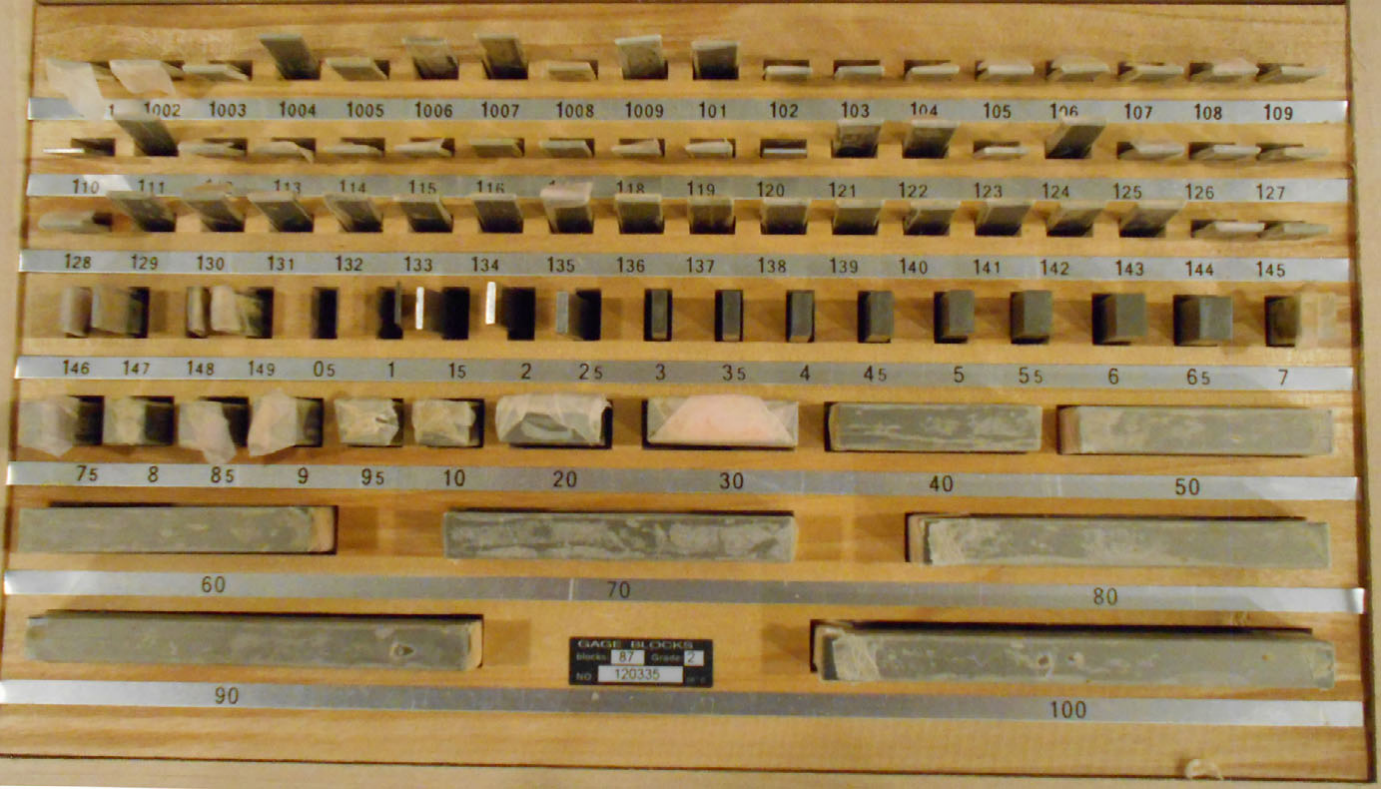
Gage blocks are readily available in sets and certifiable following the DIN681 standard. Each piece in this set has an accuracy within 0.36 μm.

**Fig 2:**
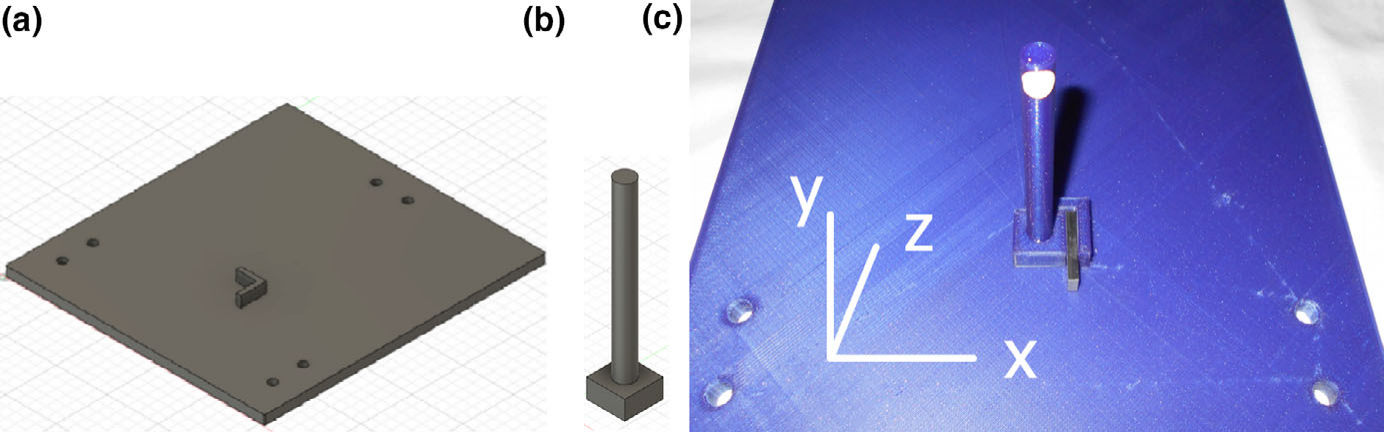
The QA tool was designed in Autodesk Fusion 360 and 3D printed. It consists of (a) a stage that fits on the mask adaptor; (b) a movable reflector post that sits against a raised corner; and (c) gage blocks that offset the reflector post by accurate distances from the corner. Offsets in x‐, y‐ and z‐directions are possible.

Our QA tool is a one‐purpose tool that uses gage blocks (Fig. 1) as a primary standard to set distances and confirm the accuracy of the HDMM readout. These blocks are readily available as DIN861‐standard certified sets with thickness accuracies <0.4 μm. The QA tool (Fig. 2) consists of a stage, which mounts on the mask adaptor and a post with a reflective marker. A raised corner on the stage center acts as a reference location for the post. The post is placed against this corner and the HDMM system zeroed. A gage block is inserted in any of the *x*, *y*, or *z* directions and the HDMM system’s measured shift compared against the block thickness. In practice, the procedure takes less than a minute to set up and confirm 1.000 mm shifts for the *x*, *y* and *z* directions. The tool is fast and effective to use and inexpensive to manufacture, all qualities desirable for a daily QA program.

## 2 MATERIALS AND METHODS

To be clear, accuracy refers to the comparison of the average reading of the HDMM system against a known standard whereas precision refers to the reciprocal of the variance. Hence a small standard deviation implies a high precision.

The QA tool was designed in Fusion 360 (AutoDesk, 2016, https://www.autodesk.com/products/fusion‐360/overview) and printed on an Ultimaker‐3 3D printer (Dynamism, 2016, https://ultimaker.com/3d‐printers/ultimaker‐3). A gage block set (Precise) was purchased. The QA tool and HDMM system were commissioned by examining the following properties:

1. Noise;
2. Stability;
3. Reproducibility;
4. Linearity; and
5. Accuracy.

### 2.A Noise

The HDMM system has inherent noise that impacts the accuracy and precision of the measurements. This test tracks the position of a stationary reflector post over a 10‐s measurement. The HDMM system's display refreshes approximately once per second. In addition to measuring the accuracy, measuring the precision is important because it impacts the accuracy and determines the minimum change in position that can be reliably detected.

### 2.B Stability

Masked treatments are on the order of 15 to 30 min long. Stability looked at the change in HDMM system's signal for a stationary reflector post over a 15‐min period. Samples were 10 s long and taken once per minute.

### 2.C Reproducibility

Setting a gap between the post and corner requires removing and reseating the post. We studied the reliability of a user reseating the post two ways. First, the HDMM system was zeroed and the post was reseated to the same position. The distribution of positions before and after was compared. Second, a paired data set was created with four different users. Each user repeated the following 10 times: (a) Zero the HDMM system and take a reading; and (b) Reseat the post and take a reading. Inter‐user variability and reproducibility were analyzed.

### 2.D Linearity

The HDMM system reports a 3D‐radial value from the origin. The linearity was tested separately on the *x*‐, *y*‐, and *z*‐axes. Negative positions were achieved by zeroing the HDMM system with a 3 mm gage block in place. The position for other blocks was *block thickness minus* 3 mm. For example, a position at (−1) mm is set with the 2‐mm gage block. Values from −3 to +3 mm were checked on each axis. The graph of the HDMM system’s reading versus position was expected to be a V‐shaped absolute value function.

### 2.E Accuracy

The average HDMM system’s position was evaluated against the position set by the gage blocks. Values for the *x*‐, *y*‐ and *z*‐axes were analyzed from the linearity data, but these were all one‐dimensional shifts and do not address the accuracy when two‐ or three‐dimensional shifts were taken. A full factorial design of experiment (DOE) was performed to capture the accuracy for shifts on one, two or three axes. Table 1 gives the parameter combinations used in the experiments. The accuracy score was $\left\{HDMM\;reading‐{\left(x^{2}+y^{2}+z^{2}\right)}^{1/2}\right\}$ and the state values for *x*, *y*, or *z* were assigned +1 for a gap of 1 mm and −1 for a gap of 0 mm. The interaction effects x*y, x*z, y*z and x*y*z describe shifts on two or three axes and have states taken as the product of the single‐variable states. If, for example, *x* = −1, *y* = −1, then x*y = (−1)(−1) = 1.

**Table 1:** The full‐factorial DOE cycles the positions through the possible state values of ±1. The interaction states were taken as the product of the x‐, y‐ and z‐states and the score was calculated by determining the difference between the nominal radial position r = (x^2^ + y^2^ + z^2^)^½^ and the HDMM system’s position.

Run	*x*	*y*	*z*	Score
1	–1	–1	–1	
2	–1	–1	1	
3	–1	1	–1	
4	–1	1	1	
5	1	–1	–1	
6	1	–1	1	
7	1	1	–1	
8	1	1	1	

## 3 RESULTS

The software packages JMP Pro 14.1.0 (SAS Institute Inc., 2018) and pro Fit 7.0.14 (Quantum Soft, 2016, Switzerland) were used to analyze the data sets.

### 3.A Noise

**Fig 3:**
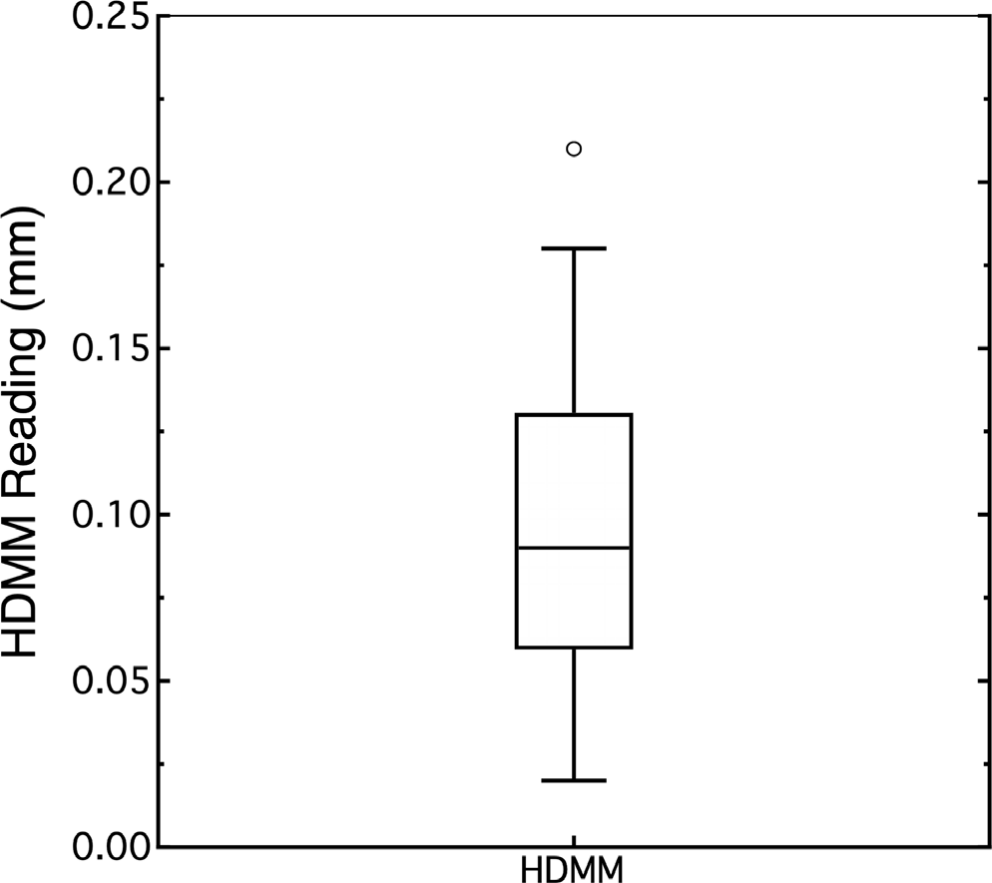
The noise of the HDMM system was evaluated using a stationary target.

Figure 3 summarizes the data in a box plot. The mean value or accuracy for the zero position was 0.099 mm (0.086, 0.111)_95%CI_ and standard deviation was 0.047 mm (0.040, 0.060)_95%CI_.

### 3.B Stability

**Fig 4:**
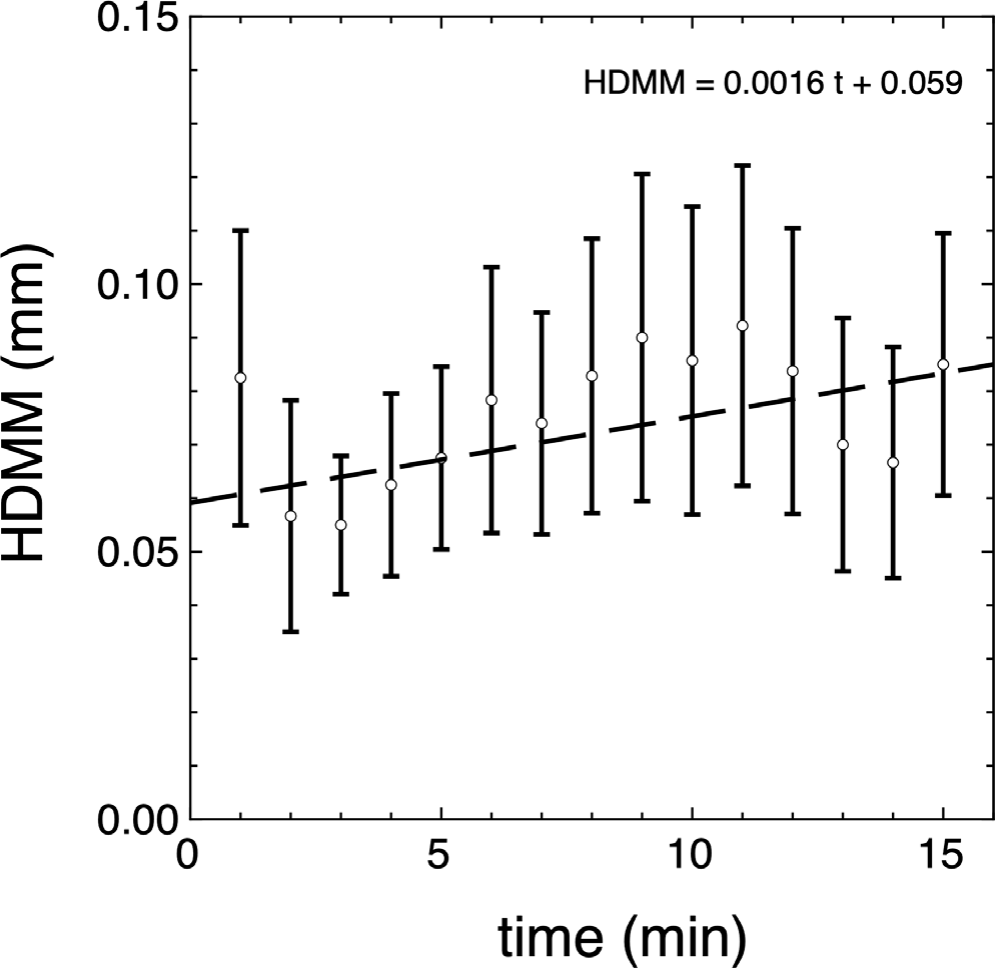
Stability reported on the average position of the reflector post, taken once per minute for 15 min. The HDMM system was zeroed on a stationary post stationary. The weighted least squares regression line and equation are shown.

Figure 4 shows the time graph of the HDMM system’s signal taken each minute over a 15‐min period. The reported drift was 0.0016 ± 0.0006 mm/min and was significantly different than zero (*P* = 0.022). The intercept was 0.059 ± 0.005 mm (*P* < 0.0001).

### 3.C Reproducibility

**Fig 5:**
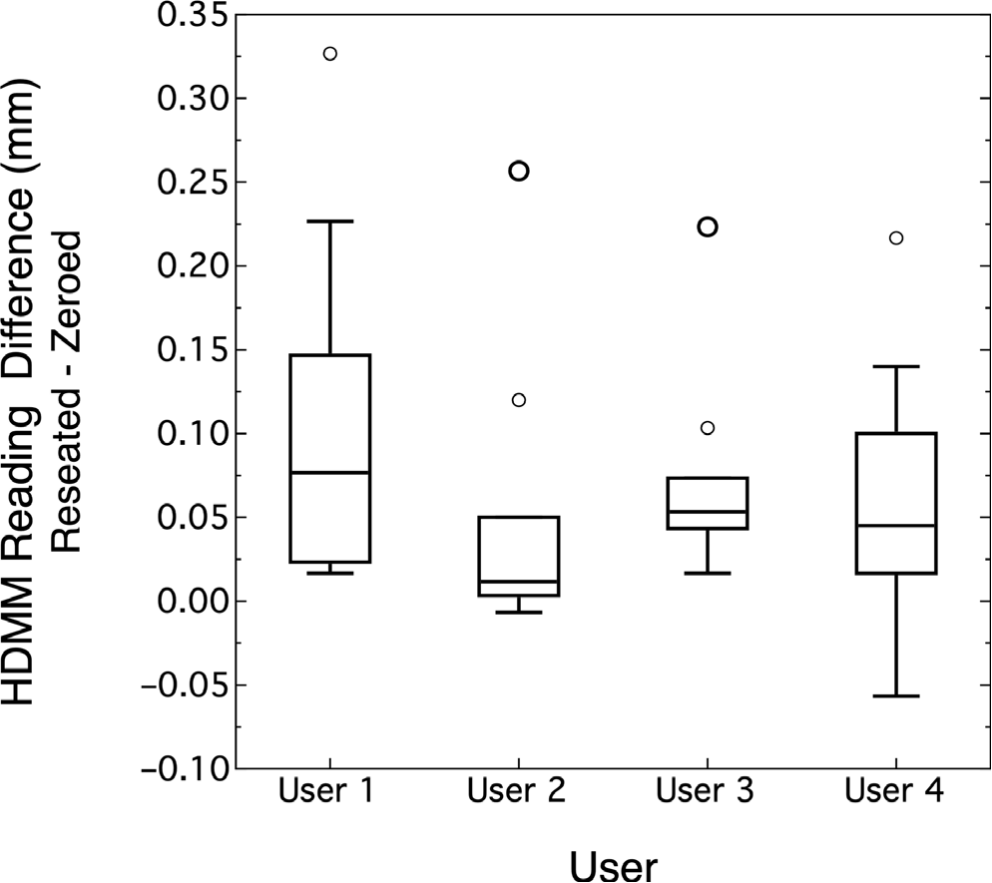
Reproducibility was evaluated by comparing the reseated reflector post to its original position. There is a 0.056 mm difference.

Figure 5 shows a box plot for repeatedly reseating the reflector post. There was a 0.056 mm (*P* < 0.0001) difference between the median values of the zeroed position and the reseated position.

**Fig 6:**
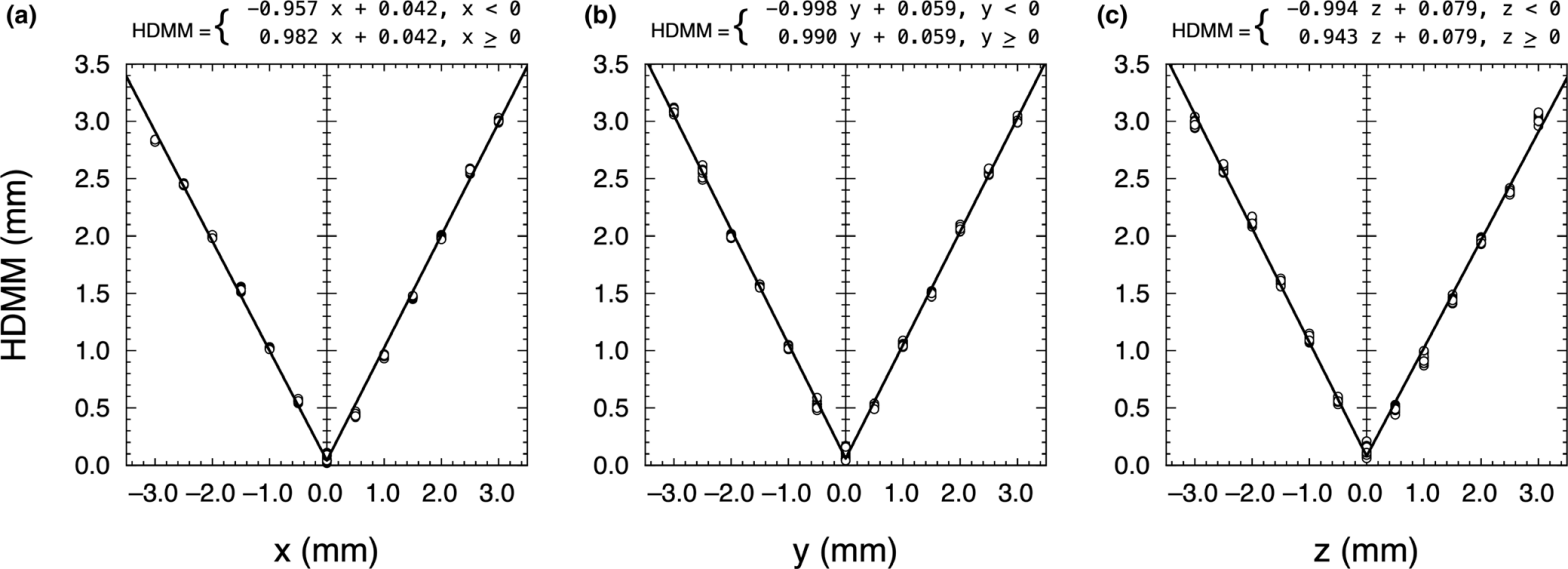
Reproducibility by user was evaluated using pair‐data sets of reseated minus zeroed positions. Each user repeated zeroing and reseating the reflector post 10 times.

Figure 6 shows the paired data results for four users. ANOVA analysis showed no significant difference between users (*P* = 0.36) and the overall average difference between reseated (after position) and zeroed (before position) is 0.072 mm (0.046, 0.098)_95%CI_.

### 3.D Linearity

**Fig 7:**
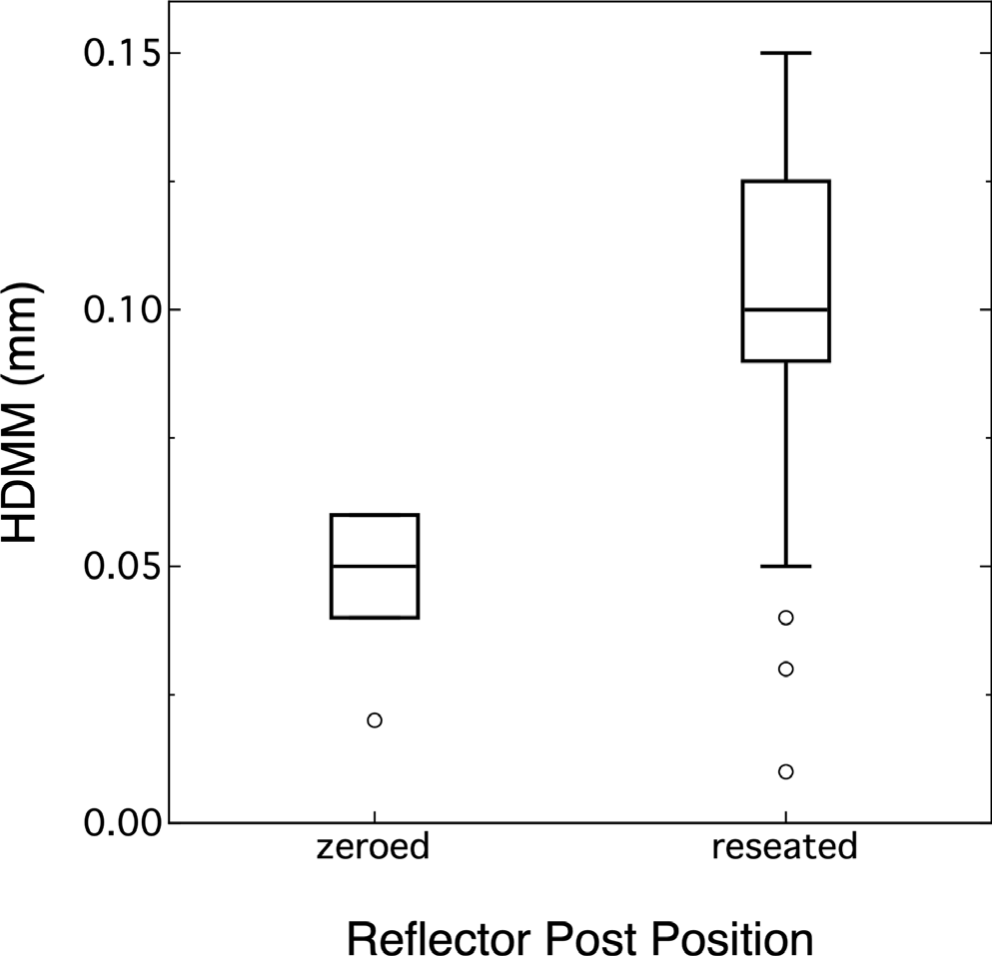
The HDMM system reports a radial distance and hence behaves as the absolute value of the displacement from zero. By setting zero with a 3 mm gage block in place, negative displacements correspond to thinner blocks and positive displacements correspond to thicker blocks

The HDMM system reports the distance from the origin and hence the readings behaved like the absolute value of the displacement in one‐dimension. The data were fitted to a bilinear model

$$HDMM=\left\{\begin{array}{cc} m_{1}x+b; & x<0\\ m_{2}x+b; & x\geq 0 \end{array}\right)$$

with similar expressions for displacements on the *y*‐ and *z*‐axes. The results are shown in Fig. 7.

### 3.E Accuracy

**Fig 8:**
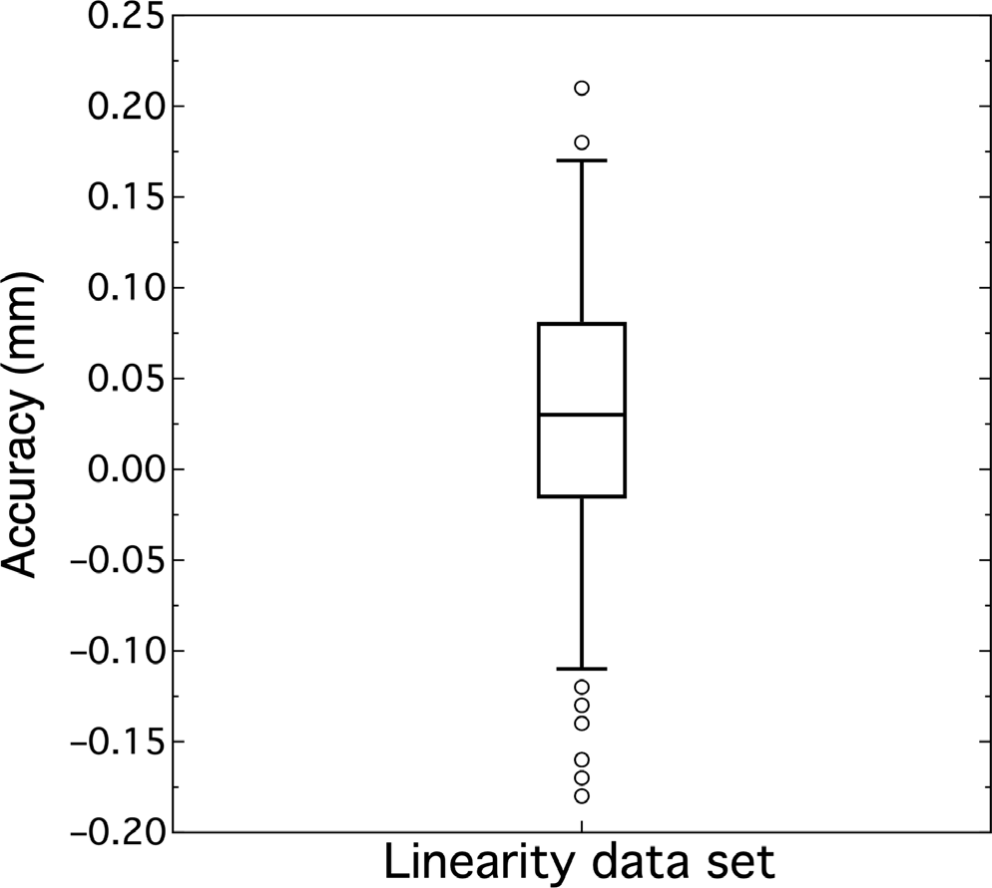
HDMM system’s accuracy from the linear data set on average is 0.026 mm with standard deviation of 0.070 mm.

The accuracy of the HDMM system against displacement can be represented as the residual of the linear model presented in the linearity study. The box plot in Fig. 8 summarizes this data, combining the results for each axis into a common data set. The overall average was 0.026 mm (0.020, 0.033)_95%CI_ with a standard deviation of 0.070 mm.

**Fig 9:**
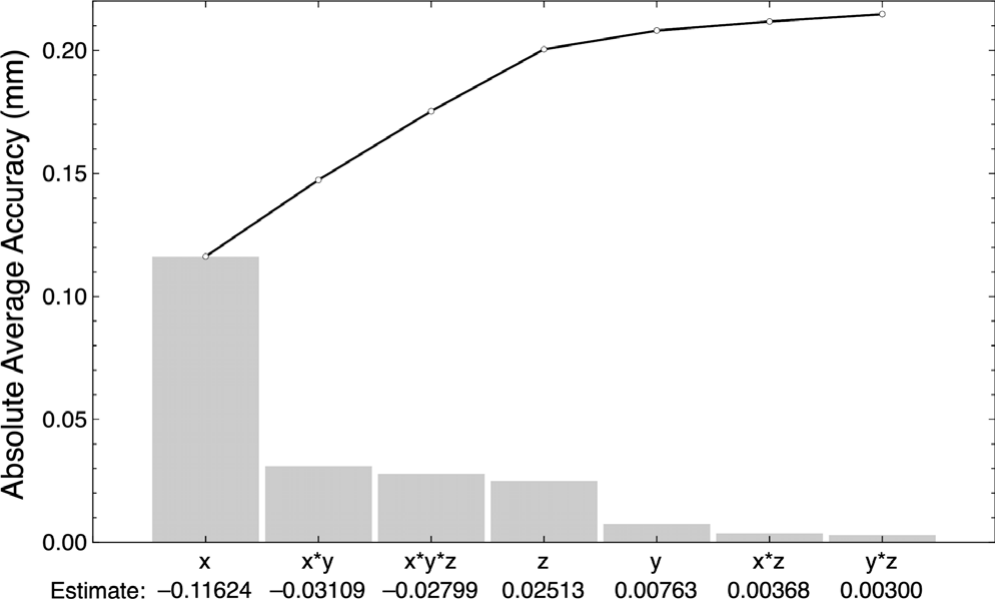
A Pareto plot showed the x‐coordinate had the greatest impact on the HDMM accuracy, while x*y, x*y*z and z were approximately equally significant, and y, x*z and y*z were the least impactful.

This was only part of the story because it does not account for full 3D motion. A full factorial DOE was performed and analyzed with the focus on identifying the variables that most strongly impact the accuracy score $HDMM‐{\left(x^{2}+y^{2}+z^{2}\right)}^{1/2}$. A Pareto plot, shown in Fig. 9, was chosen to screen for the factors important to inaccuracies in the HDMM system. The *x*‐coordinate was shown to have approximately four times greater impact on the HDMM accuracy than the next greatest effects, x*y, x*y*z and *z*. These three variables had approximately the same magnitude.

## 4 DISCUSSION

### 4.A Noise

The accuracy for a zero‐valued displacement was 0.099 mm, agreeing with accuracies stated in the literature of 0.1 mm.^2^, ^3^, ^4^ The standard deviation was 0.0407 mm, impacting the 95%CI for the mean value and setting a limit to the minimum detectable difference in signals. This minimum difference refers to how far the average value of two signals must be apart to reliably not confuse one signal for the other. Reliability involves type I and type II errors and how willing we are not to make these errors. Typically type I and type II errors are assigned α and β probabilities of 0.05, and using a single reading to estimate the average position, the minimum detectable difference^9^ is 0.26 mm. This minimum difference can be reduced by taking more readings to find the average. If one takes *N* readings, then the minimum detectable difference decreases by a factor *N* ^–½^.

### 4.B Stability

Figure 4 showed a drift of 0.0016 mm/min in the HDMM signal that was statistically significant (*P* = 0.022) but is not clinically significant. A typical masked treatment is 30 min long and according to this drift, the position of the stationary post would change by 0.048 mm. In comparison, the default threshold for treatment interruption is 1.5 mm, implying patient motion would dominate the reflector’s drift.

### 4.C Reproducibility

The reproducibility study showed there was no difference between users. Having said this, there was a small learning curve to consistently remove and replace the reflector post. Therefore, the given results were after users had some practice with this exercise. On average, the post was placed within 0.056 mm of its original position and this means the post can be accurately reseated.

### 4.D Linearity

The linearity results were close to expected values. The intercepts ranged from 0.042 to 0.079 mm and can be interpreted as the accuracy at zero displacement. The slopes were within 0.053 of the predicted ± 1 values. This can be interpreted as a relative accuracy statement of 5.3% per mm. Applied to the treatment terminating threshold of 1.5 mm, this would have an accuracy of 0.08 mm, which again is not clinically significant in comparison to the patient motion.

### 4.E Accuracy

The overall average of the linear data set was 0.026 mm, but this only looked at one‐dimensional shifts. The DOE’s Pareto plot showed the *x*‐coordinate had the greatest impact on the accuracy score. The estimate was −0.116 which described the difference between the average score for *x* in the low state (−1) and *x* in the high state (+1). Since the low state corresponds to a 0 mm position and the high state to a 1 mm position, this meant −0.058 mm per mm was the accuracy score for displacements on the *x*‐axis. It was roughly a tie between three variables for the second most significant impact. These were the *z*‐coordinate and the interaction terms x*y and x*y*z. They had approximately five times less impact than the *x*‐coordinate or about 0.013 mm per mm of displacement. Since the *x*‐coordinate had the greatest impact on the accuracy, this result showed some care is required when designing the phantom to keep the raised corner near the lateral center of the platform.

## 5 CONCLUSIONS

The HDMM system QA tool provides a good alternative to competing technologies for measuring accuracy and adds value to the QA program for the LGK Icon motion management system. It is an easy‐to‐build, simple‐to‐use and inexpensive device capable of delivering 0.1 mm accuracies. The tool sets up very quickly and all three axes can be confirmed for one mm shifts in less than two minutes, making it suitable for daily QA use.
